# PDE4 Inhibition Reduced Osteoclast Differentiation in Psoriatic Patients

**DOI:** 10.3390/life15030467

**Published:** 2025-03-14

**Authors:** Annunziata Raimondo, Alessia Balestrino, Serena Lembo

**Affiliations:** Department of Medicine Surgery and Dentistry, “Scuola Medica Salernitana”, University of Salerno, 84084 Salerno, Italy; abalestrino@unisa.it (A.B.); slembo@unisa.it (S.L.)

**Keywords:** psoriasis, inflammation, apremilast

## Abstract

Background: Psoriatic skin inflammation has been linked to joint inflammation and bone structural alterations, contributing to a “pro-osteoclastogenic march.” Osteoclasts (OCs), responsible for bone resorption, originate from monocytes/macrophages and are regulated by the RANKL-RANK signaling pathway. The cyclic AMP (cAMP) pathway plays a crucial role in OC maturation, and phosphodiesterases (PDEs) control its intracellular levels. Apremilast, a selective PDE4 inhibitor used for psoriasis (Pso) and psoriatic arthritis (PsA) treatment, may modulate osteoclastogenesis. Methods: Seventeen patients with moderate-to-severe psoriasis without arthritis, eligible for systemic apremilast therapy, were enrolled. Blood samples were collected at baseline and after 52 weeks of treatment to evaluate in vitro osteoclastogenesis from peripheral blood monocytes/macrophages and to measure serum RANKL levels. Results: After 52 weeks of apremilast treatment, OC and RANKL levels were significantly reduced in psoriatic patients compared to baseline. A sub-analysis was performed on two age- and sex-matched subgroups: a bionaïve group and a bioexperienced group. Bioexperienced patients exhibited lower OCP counts and reduced plasma RANKL levels compared to bionaïve patients. Conclusions: Our findings highlight the role of PDE4 in the pro-osteoclastogenic process in psoriasis and suggest that apremilast may counteract bone resorption by modulating RANKL levels and osteoclast differentiation, with potential clinical implications.

## 1. Introduction

Psoriasis (Pso) is a chronic inflammatory skin disease that affects approximately 2–3% of the global population and is associated with multiple systemic comorbidities. Moreover, psoriatic arthritis (PsA) often arises as a consequence of psoriasis, representing another clinical manifestation of the disease [1,2]. The proportion of patients with psoriasis who develop PsA remains controversial, with estimates ranging from 6% to 42% depending on the population studied [3]. PsA has a highly variable and unpredictable course, ranging from mild, non-destructive forms to severe, erosive, and deforming disease. The risk of developing PsA is positively correlated with the severity of skin involvement and is higher when psoriasis affects certain anatomical sites considered predictive, such as the nails, scalp, and intergluteal–sacroiliac region. Notably, cutaneous manifestations precede the onset of joint involvement by an average of 12 years in approximately 85% of cases [4,5].

Previous studies have demonstrated that psoriatic skin inflammation contributes to joint inflammation and subsequent bone structural damage in a process referred to as the “pro-osteoclastogenic march” [6].

At the pathophysiological level, bone erosion in PsA is primarily driven by osteoclasts (OCs), multinucleated cells responsible for bone resorption that originate from the monocyte/macrophage lineage. A key regulatory pathway in bone resorption is the receptor activator of nuclear factor-kappa B ligand (RANKL) and its receptor (RANK), which play a pivotal role in osteoclast differentiation and activation [7].

OC maturation is a complex process involving multiple intracellular signaling pathways, including the cyclic adenosine monophosphate (cAMP) pathway, which induces RANKL expression in osteoblasts. Intracellular cAMP levels are regulated by the balance between its synthesis, mediated by adenylate cyclase, and its degradation, controlled by phosphodiesterases (PDEs) [8].

Several small-molecule PDE inhibitors have been studied to determine their role in bone metabolism and are considered promising candidates for the pharmacological treatment of osteoporosis [9]. However, their precise role in osteoclastogenesis remains incompletely understood. Apremilast, a selective PDE4 inhibitor and the primary isoform expressed in inflammatory cells, is currently approved for the treatment of Pso and PsA [10]. Both keratinocytes and osteoclasts are key target cells in this approach. Apremilast exerts its effects on keratinocytes by inhibiting the production of pro-osteoclastogenic cytokines, thereby reducing signals that promote osteoclast differentiation. Simultaneously, it directly impacts osteoclasts by inhibiting their maturation and differentiation through the reduction in RANKL levels. Additionally, its anti-inflammatory action further contributes to limiting osteoclastogenesis, highlighting its dual mechanism in the context of PsA-related bone erosion.

This study aimed to investigate the effects of apremilast therapy on the osteoclastogenesis process in patients with moderate-to-severe psoriasis. To this end, we enrolled patients with moderate-to-severe psoriasis without clinical signs of arthritis who were candidates for systemic therapy with apremilast.

## 2. Materials and Methods

### 2.1. Study Population

A total of 17 adult patients with moderate-to-severe psoriasis (9 males, 8 females; mean age 61 ± 14 years) who were eligible for apremilast therapy, along with 15 healthy controls (7 males, 8 females; mean age 54 ± 24 years), were enrolled (Table 1). The study was conducted in accordance with the Declaration of Helsinki, and all participants provided written informed consent before enrollment. The study protocol was reviewed and approved by the Ethics Committee for Clinical Studies (CECS), with approval number 38_r.pso (session date: 17 February 2021). Blood samples were collected at baseline (W0) and after 52 weeks (W52) of apremilast treatment. Disease assessment was performed at baseline and W52, evaluating the percentage change (Δ%W52/W0) in the Psoriasis Area Severity Index (PASI), Body Surface Area (BSA), Dermatology Life Quality Index (DLQI), and Nails and Scalp Physician Global Assessment (PGA). A sub-analysis was performed on two age- and sex-matched subgroups of psoriatic patients: a bionaïve group, consisting of 5 patients (3 males, 2 females) with a mean PASI of 15 who had never received biologic treatments, and a bioexperienced group, composed of 5 patients (3 males, 2 females) with a mean PASI of 12 who had previously been treated with at least two different biologic drugs, primarily from the anti-TNF and anti-IL-17 classes, for an average of 4 ± 2.2 years. Inclusion criteria required a diagnosis of moderate-to-severe plaque psoriasis for at least 12 months before the screening visit. The exclusion criteria included a history of other inflammatory skin diseases, PsA, infections, autoimmune disorders, systemic diseases, or conditions leading to immunosuppression or malignancy. The details are reported in Table 1.

### 2.2. Peripheral Blood Mononuclear Cell (PBMC) Purification and Osteoassay

Peripheral blood samples were collected from 17 psoriatic patients at baseline (W0) and after 52 weeks (W52) of treatment, as well as from 15 healthy controls. The samples were diluted 1:1 with sterile Hank’s Balanced Salt Solution (HBSS, YourSial, Rome, Italy; Cat.n SIAL HBSS-2) and layered over Ficoll-Paque Plus (Cytiva, Marlborough, MA, USA; Cat.n 17-1440-02) at room temperature. Following centrifugation at 400× *g* for 30 min, the peripheral blood mononuclear cell (PBMC) fraction was carefully isolated. The collected PBMCs were then washed twice with phosphate-buffered saline (PBS, YourSial, Rome, Italy; Cat.n SIAL-PBS-1A) and resuspended in a complete culture medium for subsequent analyses. Monocytes/macrophages were isolated from the PBMCs using the adherence method in a 96-well plate for an in vitro assessment of spontaneous osteoclastogenesis. The specificity of the isolated monocytes was confirmed by fluorescence-activated cell sorting (FACS) analysis using an anti-CD14 monoclonal antibody (Miltenyl-biotech human CD14-FITC Clone TÜK4, Auburn, CA, USA; Cat. n 130-080-701). Prior to differentiation, the PBMCs from both W0 and W52 patients were counted, and cell viability was assessed using Trypan Blue. To induce osteoclast differentiation, the culture medium was supplemented with recombinant human macrophage colony-stimulating factor (M-CSF, 25 ng/mL, Cell Guidance Systems Ltd., Cambridge, UK; Cat. n GFH3-100) and recombinant human receptor activator of nuclear factor kappa-B ligand (RANKL, 50 ng/mL, Cell Guidance Systems Ltd., Cambridge, UK; Cat. n GFH19AF-100). The differentiation medium was refreshed every 2–3 days for a total of 14 days.

Osteoclast precursors (OCPs) were identified by staining for tartrate-resistant acid phosphatase (TRAP; Cosmo Bio, Carlsbad, CA, USA; Cat.No.PMC-AK04F-COS), following the manufacturer’s instructions. TRAP-positive multinucleated cells (≥3 nuclei) were counted using light microscopy and analyzed with ImageJ software (version 1.54m) [6].

### 2.3. ELISA

The plasma levels of RANKL were quantified in both psoriatic patients and healthy controls using an enzyme-linked immunosorbent assay (ELISA) kit (Fine test, Wuhan, China, Cat. n EH0313). Undiluted plasma samples were directly added to a 96-well plate pre-coated with an anti-RANKL antibody. After incubation, unbound components were removed by washing, and a biotin-conjugated anti-RANKL detection antibody was added, followed by HRP-Streptavidin. The reaction was visualized using TMB substrate, and absorbance was measured at 450 nm with an Infinite M200 PRO plate reader (Tecan, Grödig, Austria). The concentration of RANKL in the samples was determined by generating a standard curve.

### 2.4. Statistical Analysis

All experiments were performed in triplicate. Data are presented as the mean ± standard deviation (SD). The statistical analysis was conducted using GraphPad Prism 6.0. The normality of data distribution was assessed using the Shapiro–Wilk test. Comparisons between baseline (W0) and post-treatment (W52) within the same group were performed using a paired *t*-test (#). The differences between the psoriatic patients and healthy controls, as well as between the bionaïve and bioexperienced subgroups, were analyzed using the Mann–Whitney U test (*). Statistical significance was set at * *p* < 0.05; ** *p* < 0.01.

## 3. Results

At baseline, the mean PASI was 11.50, BSA was 16.20%, DLQI was 18, and Nails and Scalp PGA was 3. After 52 weeks of apremilast treatment, PASI decreased to 1.50, BSA to 3%, DLQI to 0.5, and Nails and Scap PGA to 1. This corresponds to a 73% reduction in PASI, an 86% decrease in BSA, and an 83% improvement in DLQI. Additionally, Nails and Scalp PGA showed a 33.3% improvement (Table 1).

**Figure 1:**
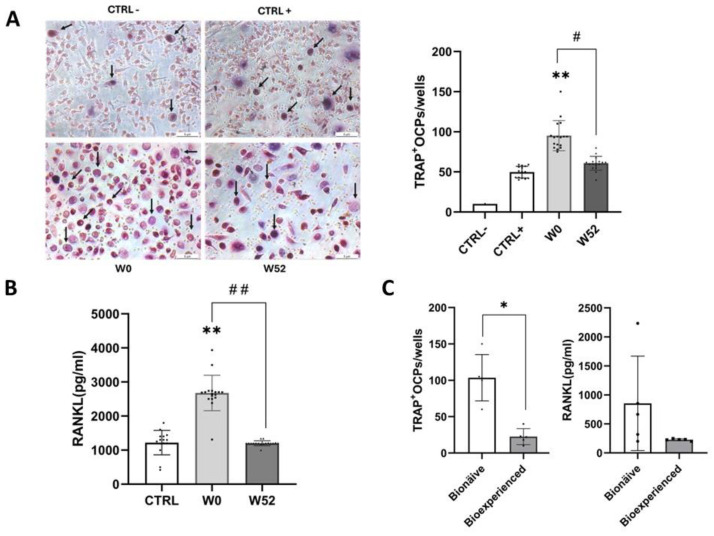
Effects of apremilast on osteoclast precursors and plasma RANKL levels in psoriatic patients. (**A**) Osteoclast precursor (OCP) number in psoriatic patients without PsA (*n* = 17) before (W0) and after 52 weeks (W52) of apremilast with respect to healthy controls (*n* = 15). OCP was calculated by counting tartrate-resistant acid phosphatase (TRAP)-positive cells with 3 or more nuclei/well, indicated by representative black arrows. (**B**) RANKL plasma levels in psoriatic patients without PsA before (W0) and after 52 weeks (W52) of apremilast. (**C**) Differences in OCP number and RANKL plasma levels in bioexperienced (*n* = 5) and bionaïve (*n* = 5) enrolled patients after 52 weeks of treatment. Data are shown as mean ± SD. Statistical differences between W52 and W0 (#) were calculated using paired *t*-test; statistical differences between psoriatic patients and CTRLs, as well as bioexperienced and bionaïve groups (*), were calculated using Mann–Whitney test. * *p* < 0.05; ** *p* < 0.01; # *p* < 0.05, ## *p* < 0.01. CTRL-, monocytes from healthy controls without growth factor (RANKL and M-CSF); CTRL+, monocytes from healthy controls with growth factor (M-CSF 25 ng/mL, RANKL, 50 ng/mL); W0, monocytes from psoriatic patients at baseline with growth factor (M-CSF 25 ng/mL, RANKL, 50 ng/mL); W52, monocytes from psoriatic patients after 52 weeks of treatment with apremilast with growth factor (M-CSF 25 ng/mL, RANKL, 50 ng/mL).

PBMC viability was assessed after 52 weeks of apremilast treatment, with no significant differences observed between baseline (W0, monocytes from psoriatic patients at baseline cultured with M-CSF (25 ng/mL) and RANKL (50 ng/mL)) and post-treatment (W52, monocytes from psoriatic patients after 52 weeks of apremilast treatment cultured with growth factors). The number of osteoclast precursors (OCPs) was significantly higher in psoriatic patients compared to the healthy controls (CTRL+; *p* = 0.0017), where CTRL+ refers to monocytes from the healthy controls cultured with M-CSF and RANKL, and CTRL− represents monocytes from the healthy controls cultured without growth factors. Notably, after 52 weeks of treatment (W52), OCP levels were significantly reduced compared to baseline (W0) (*p* = 0.003) (Figure 1A).

Similarly, plasma RANKL levels were significantly elevated in psoriatic patients compared to the healthy controls (*p* < 0.001). However, PDE4 inhibition with apremilast led to a significant reduction in plasma RANKL levels (*p* = 0.0025) compared to baseline (W0), reaching levels similar to those of the healthy controls. (Figure 1B).

A sub-analysis was performed on two age- and sex-matched subgroups of psoriatic patients after 52 weeks of treatment with apremilast: a bionaïve group (*n* = 5; 3 males and 2 females; mean PASI: 15) and a bioexperienced group (*n* = 5; 3 males and 2 females; mean PASI: 12). Bioexperienced patients exhibited lower OCP counts and reduced plasma RANKL levels compared to bionaïve patients (Figure 1C).

## 4. Discussion

Our findings offer new insights into the role of PDE4 inhibition in regulating osteoclastogenesis in patients with moderate-to-severe psoriasis. In this study, we confirmed that OCPs and plasma RANKL levels were significantly elevated in psoriatic patients compared to the healthy controls, reinforcing the concept of a “pro-osteoclastogenic march” in psoriasis. Notably, after 52 weeks of apremilast treatment, both OCP counts and RANKL levels showed a significant reduction. These results suggest that PDE4 inhibition may help counteract the osteoclastogenic process, potentially mitigating bone resorption and structural damage in psoriatic patients.

The involvement of the RANKL-RANK signaling pathway in osteoclast differentiation is well established, with RANKL playing a key role in osteoclast activation. The reduction in RANKL levels following apremilast treatment suggests that PDE4 inhibition may indirectly influence osteoclast differentiation by modulating upstream inflammatory pathways. Previous studies have established that PDE4 inhibitors suppress pro-inflammatory cytokines such as TNF-α, IL-6, and IL-17, all of which are known to contribute to osteoclast activation and bone resorption [11]. Our findings align with these reports, supporting the idea that apremilast may impact bone metabolism through its anti-inflammatory effects. Additionally, our sub-analysis of bioexperienced and bionaïve patients revealed lower OCP counts and reduced RANKL levels in the bioexperienced group. These preliminary data could highlight that early intervention with target therapies may interfere with the disease course and progression [12,13]. However, this hypothesis needs further study. It is worth noting that, to date, only one in vitro study has investigated the effect of apremilast on osteoclastogenesis using PBMCs from patients with Pso, Pre-PsA, and PsA and healthy controls treated with APR 10 µM added to culture medium [14]. Our study investigated osteoclast differentiation in patients with moderate-to-severe psoriasis without PsA treated with apremilast for 52 weeks in a real-world setting. The data were compared with those from Pso patients before the treatment and with those from healthy controls. Moreover, RANKL plasma levels were assessed in our study, highlighting the novel effect of apremilast on pro-osteoclastogenic factors. Our results provide clinically relevant evidence for the role of PDE4 in bone metabolism. However, this study has some limitations. The relatively small sample size and single-center design may limit the generalizability of our findings. Additionally, our analysis focused specifically on patients with Pso without PsA to better understand the contribution of cutaneous inflammation to osteoclastogenesis.

## 5. Conclusions

Taken together, our data highlight the role of PDE4 in the pro-osteoclastogenic process in psoriasis and suggest that apremilast may counteract bone resorption by modulating RANKL levels and osteoclast differentiation, with potential clinical implications.

## Figures and Tables

**Table 1:** Demographic and clinical characteristics of enrolled patients.

Characteristic	Psoriatic Patients (*n* = 17)	Control Group (*n* = 15)	*p*-Value
Male (%)	9 (52.9)	7 (46.6)	
Female (%)	8 (47)	8 (53.3)	
Age, mean (SD), y	61 (±14)	54 (±24)	
PASI, mean (SD)	11.50 (±7.91)	-	
BSA, mean % (SD)	16.20 (±0.15)	-	
DLQI, mean (SD)	18 (±7.94)	-	
Nails PGA, mean (SD)	3 (±1)		
Scalp PGA, mean (SD)	3 (±1)		
	Disease severity index improvement Δ%W52/W0		
PASI	−73%		
BSA	−86%		
DLQI	83%		
Nails PGA	33.3%		
Scalp PGA	33.3%		
	Bioexperienced group (*n* = 5)	Bionaïve group (*n* = 5)	
Male Female Age, mean (SD), y PASI, mean (SD) Previous biological Therapy (*n*)	3 2 53 (±7) 12 ± 4 Anti TNF-α (3) Anti il-17A (3)	3 2 55 (±6) 15 ± 3 - -	

PASI, Psoriasis Area Severity Index; BSA, Body Surface Area; DLQI, Dermatology Life Quality Index; PGA, Physician Global Assessment; SD, standard deviation.

## Data Availability

The data supporting this study’s findings are available from the corresponding author upon request.

## Abbreviations

The following abbreviations are used in this manuscript:

Pso	Psoriasis
PsA	Psoriatic arthritis
OCs	Osteoclasts
RANKL	Receptor activator of nuclear factor-kappa B ligand
RANK	Receptor activator of nuclear factor-kappa B
cAMP	Cyclic adenosine monophosphate
PDEs	Phosphodiesterases
PDE4	Phosphodiesterase 4
PASI	Psoriasis Area Severity Index
BSA	Body Surface Area
DLQI	Dermatology Life Quality Index
PGA	Physician Global Assessment
PBMCs	Peripheral Blood Mononuclear Cells
HBSS	Hank’s Balanced Salt Solution
PBS	Phosphate-buffered saline
FACS	Fluorescence-activated cell sorting
M-CSF	Macrophage colony-stimulating factor
OCPs	Osteoclast precursors
TRAP	Tartrate-resistant acid phosphatase
ELISA	Enzyme-linked immunosorbent assay
SD	Standard deviation
